# A survey on critical health competences among diabetes educators using the Critical Health Competence Test (CHC Test)

**DOI:** 10.1186/s12909-021-02519-9

**Published:** 2021-02-09

**Authors:** Lars Hecht, Gabriele Meyer, Anke Steckelberg

**Affiliations:** 1 School of Nursing Science, Faculty of Health, University of Witten/Herdecke, Witten, Herdecke Germany; 2 Institute for Health and Nursing Science, Medical Faculty, Martin Luther University Halle-Wittenberg, Halle (Saale), Germany

**Keywords:** Critical health competence, Critical health literacy, Evidence-based medicine, Evidence-based practice, Diabetes, Diabetes educator, Shared decision-making

## Abstract

**Background:**

Diabetes associations claim to have a patient-centered approach in diabetes care including shared decision-making (SDM). Diabetes educators are important healthcare professionals for implementing the concept of informed SDM in diabetes care. They need critical health competences (CHC) in order to provide evidence-based information and to support patients in understanding the risks of the disease and also the possible benefits or harm of the healthcare options. Therefore, we surveyed the CHC of diabetes educators.

**Methods:**

We performed a cross-sectional survey using the validated Critical Health Competences (CHC) Test to measure CHC of certified diabetes educators and trainees in Germany. Diabetes educators were approached via newsletter, mailing lists or in person during the conference of the German Diabetes Association. Trainees were approached during their training sessions. We applied scenario 1 of the CHC test, which comprises 17 items with open-ended and multiple-choice questions. Mean person parameters with a range from 0 to 1000 were calculated to assess the levels of critical health competences and a multiple linear regression analysis was conducted to determine correlations between sociodemographic variables and levels of CHC.

**Results:**

A total of 325 participants, mean age 38.6 (±11.1) years, completed the CHC test; *n* = 174 (55.5%) were certified diabetes educators and *n* = 151 (46.5%) were trainees. The participants achieved a mean score of 409.84 person parameters (±88.10) (scale from 0 to 1000). A statistically significant association was found only between the level of education and the level of CHC (b = 0.221; *p*-value 0.002). Participants with grammar school education achieved higher mean scores compared to participants with secondary school education (432.88 ± 77.72 vs. 396.45 ± 85.95; mean difference 36.42 ± 9.29; 95%CI 18.15 to 54.71; *p* < 0.0001).

**Conclusion:**

Diabetes educators achieved low competence scores and it can be assumed that they do not have sufficient CHC to conduct consultations based on the SDM principles. Poor CHC among healthcare providers are a major barrier for the implementation of SDM. Core concepts of evidence-based medicine should be implemented into the curricula for diabetes educators in order to increase their levels of CHC.

## Background

Patients increasingly prefer active roles in healthcare decision-making. They want to be involved in health-related decisions, regardless of their current medical condition, age or sex [1–5]. Health authorities have highlighted the need to involve patients in the development and supply of healthcare, and request informed shared decision-making (SDM), where patients and healthcare providers simultaneously participate by sharing information and jointly negotiating the treatment options [6, 7]. In the informed SDM process, evidence-based information, including benefits and harm of all treatment options, has to be provided and patients’ values and preferences have to be clarified. The healthcare professional needs at least to explain the treatment options and their possible consequences for the patient [8, 9]. As a result, an informed choice can be made which is based on relevant knowledge, and the decision-maker’s attitude is consistent with the chosen option [8]. However, several barriers in the implementation of informed shared decision-making in clinical practice have been identified. Structural factors such as lack of time as well as a power imbalance in the relationship between the patient and the healthcare professional have been stated [10, 11]. Another major barrier is found in the poor CHC among healthcare providers [12]. The World Health Organizations´ healthy schools initiative defines health literacy as “… the cognitive and social skills, which determine the motivation and ability of individuals to gain access to understand and use information in ways which promote and maintain good health” [13]. Health literacy is a complex construct with different dimensions and it has been defined in multiple ways. It concerns the motivation, knowledge and competences a person needs to understand, appraise and apply health-related information to improve their own health [14]. The well-established model of health literacy by Nutbeam distinguishes between three levels of health literacy:functional -, interactive -, and critical health literacy. Critical health literacy includes higher level of cognitive and social skills required to critically appraise different information and to engage in informed SDM [15]. So, a high level of CHC can enhance patient autonomy with regard to therapeutic decisions.

In the field of diabetes mellitus, informed SDM plays an important role. People with diabetes mellitus are subjected to many behavioral recommendations, such as adhering to dietary and medication prescriptions or concrete advice for reducing their bodyweight. The American Diabetes Association (ADA) and the European Association for the Study of Diabetes (EASD) claim a “patient-centered approach” in the care of people with diabetes. These approaches explicitly include informed SDM in diabetes therapy [16].

In Germany, diabetes educators could play a pivotal role in implementing the concept of informed SDM. Diabetes educators are healthcare professionals with a nursing or nutritional background who teach patients with diabetes mellitus to manage their condition and support them in doing so. As a prerequisite for providing informed SDM, diabetes educators need competences to critically appraise and appropriately communicate scientific information. Training to become a diabetes educator comprises 1800 h of partly theoretical and partly practical education [17]. However, the concept of evidence-based medicine is not consistently part of the training. Former studies suggested that diabetes educators overestimate the effects of therapeutic interventions when results of clinical trials are promoted in a biased way, and risk literacy is still low among this group of healthcare professionals [18]. Hence, we aimed to survey CHC in diabetes educators. To measure CHC of diabetes educators we used the validated CHC test developed by Steckelberg et al. [19].

## Methods

### Study design

The cross sectional survey aimed to measure CHC of specialized healthcare professionals.

### Participants

Eligible participants for the survey were certified diabetes educators working in outpatient or inpatient settings and trainees. Healthcare professionals, who belonged to one of these groups and who expressed willingness, were invited to participate in the study.

### Recruitment

The recruitment comprised three different strategies. First, announcements were published via newsletters and mailing lists. If one of the addressed healthcare professionals responded and expressed interest, the questionnaire was sent to the potential participant via email. Second, diabetes educators attending the conference of the German Diabetes Association in May 2018 were invited to participate. Third, trainees were contacted during their training sessions held in a training facility of the German Diabetes Association and invited to participate. These trainees had just started their 1800 h of theoretical and practical training. They were invited to complete the questionnaire during one of their training sessions Recruitment was carried out between October 2017 and February 2019.

### Outcomes

The primary outcome was CHC, as assessed by the CHC-test [19]. The secondary outcomes were correlations between sociodemographic variables and levels of critical health competences and corresponding differences in levels of competence.

The CHC-test is a feasible and valid instrument for measuring critical health competences and comprises different content areas of health care. These topics are transformed into scenarios resulting in items. The items cover major competences and skills that belong to the critical health literacy construct: understanding of medical concepts, literature search skills as well as comprehension of basic statistics and study designs.

The complete CHC-test includes four medical scenarios resulting in four test sheets with a total of 72 items. Reliability (WINMIRA ANOVA) across all items is 0.88 and for the single scenarios 0.74 (scenario 1); 0.68 (scenario 2); 0.69 (scenario 3) and 0.74 (scenario 4) [19].

Open-ended and multiple-choice questions were evenly distributed within the test. Completion of the all four test sheets did not exceed 90 min.

However, single scenarios can be applied to measure CHC avoiding overload of the tested persons and increasing study participation.

To evaluate the level of CHC, person parameters are calculated with a range from 0 to 1000. Higher person parameters reflect a higher level of CHC.

### Data collection

In the present study, we applied scenario 1 with 17 items [19]. We chose scenario 1, since the items of the first scenario were the simplest. Therefore, less competence (lower person parameters) is needed to solve the items of scenario 1. In order to achieve comparability of person parameters, we adjusted for different scenario difficulties.

Sociodemographic characteristics including sex, age, country of birth, level of education, profession and completed professional training in the field of health science were collected.

Diabetes educators who had been recruited via announcement returned the completed questionnaire via mail or email. Diabetes educators attending the conference of the German Diabetes Association in May 2018 were asked to fill in the questionnaire at the exhibition stand of the German Association of Diabetes Educators. Data collection for the trainees took place in three certified training centers run by the German Diabetes Association at the University of Applied Science of Rheine, the University of Jena and the Health Academy of Regensburg. Trainees were invited to complete the questionnaire during one of their training sessions.

### Data analysis

DB and DA entered all the data in SPSS Version 21 according to the coding instruction. To ensure objective analyses, the coding rules for open-ended questions and the scoring rules for multiple choice items were applied. AS trained the coders to stick to the coding instructions and LH checked the data for accuracy. The baseline characteristics were analyzed descriptively. When applicable, means and SDs were calculated. Levels of CHC were assessed by calculating mean person parameters with a range from 0 to 1000. A multiple linear regression analysis was performed to determine the correlations between sociodemographic variables and levels of CHC. Through the multiple linear regression analysis, it was checked whether sex, age, country of birth, level of education, profession and completed professional trainings in the field of health science are significantly associated with the levels of CHC. Mean person parameters were included as the dependent continuous variable. A *p* value of < 0.05 was considered statistically significant. The Student’s t-test was applied to examine the differences in the levels of CHC among the relevant sociodemographic variables. Corresponding 95% CIs were calculated to assess the range of differences in levels of competence.

### Ethical considerations

The participants were informed about anonymized data collection and analysis. All the participants were free to answer the questions and all the diabetes educators in training were told that the survey was anonymous and had no impact on their grading.

The study was approved by the ethical committee of the Medical Faculty of the Martin Luther University Halle-Wittenberg (reference number 2017–75).

## Results

### Participants

A total of 325 participants, mean age 38.6 (±11.1) years, took part in the study and completed the questionnaires. The majority of participants was born in Germany. Only 21 participants originated from other countries and had lived in Germany for 21.6 (±11.8) years. The majority (*n* = 197) stated German as their native language, 17 participants had another native language and 111 did not respond to this question. The participants´ average professional experience was 12.9 (±11.1) years. Approximately half of the participants (46.2%) had completed advanced training in various fields of medicine; for example, study nurse or stroke nurse.

Table 1 displays the descriptive characteristics of the participants.

**Table 1:** Participants characteristics

Sex (***n*** = 320)	Female	303 (94.7%)^a^
	Male	17 (5.3%)
**Age**	38.6 (±11.1) years	
**Training status (*n*** **= 325)**	Certified diabetes educator	174 (53.5%)
	Trainee	151 (46.5%)
**Level of education**^**b**^ **(*n*** **= 316)**	Secondary school	176 (55.5%)
	Grammar school	140 (44.3%)
**Basic profession (*n*** **= 322)**	State-examined nurse, 3 yrs. vocational training	106 (32.9%)
	Medical assistant	84 (26.1%)
	Dietician	83 (25.8%)
	Nutritional scientist	30 (9.3%)
	More than one basic profession	10 (3.1%)
	Physical therapist	4 (1.2%)
	Medical technical assistant	2 (0.6%)
	Pharmaceutical technical assistant	1 (0.3%)
	Midwife	1 (0.3%)
	Sports scientist	1 (0.3%)
**Country of origin (*n*** **= 317)**	Germany	296 (93.4%)
	Russia	5 (1.6%)
	Poland	4 (1.3%)
	Turkey	4 (1.3%)
	Kazakhstan	2 (0.6%)
	Austria	2 (0.6%)
	Switzerland	1 (0.3%)
	Serbia	1 (0.3%)
	Greece	1 (0.3%)
	Portugal	1 (0.3%)
**Native language (*n*** **= 214)**	German	197 (91.6%)
	Turkish	5 (2.3%)
	Polish	5 (2.3%)
	Russian	3 (1.4%)
	Serbian	1 (0.5%)
	Spanish	1 (0.5%)
	Tamil	1 (0.5%)
	Portuguese	1 (0.5%)
**Professional experience (*n*** **= 302)**	12.6 (±11.1) years	
**Participants with completed advanced training in various fields of medicine (*n*** **= 316)**	146 (46.2%)	

^a^Percentages may not total 100 due to rounding

^b^ Grammer school education includes the diploma corresponding to university entrance level, whereas secondary school education does not

### Critical health competences

The achieved mean score across all participants was 409.84 (±88.10). A statistically significant association was found only between the level of education and the level of CHC. The regression coefficient for the level of education was 0.2 (*p*-value 0.002), reflecting higher levels of CHC for higher levels of education. Subjects with grammar school education achieved a slightly higher mean score of person parameters compared to participants with secondary school education (432.88 ± 77.72 vs. 396.45 ± 85.95, mean difference 36.42 ± 9.29; 95%CI 18.15 to 54.71; *p* < 0.0001). No differences were found in the levels of CHC among the relevant sociodemographic variables under study (Table 2).

**Table 2:** Differences in the levels of CHC for the relevant sociodemographic variables under study

	Mean person parameters	Mean difference	95% CI	*P*-value
Training status	Trainee (n = 174) 414.44 ± 71.50	9.89 ± 10.05	−9.38 to 29.18	0.33
	Diabetes educators (n = 151) 404.54 ± 104.01			
Level of education	Grammar school (*n* = 142) 432.88 ± 77.72	36.42 ± 9.29	18.15 to 54.71	< 0.0001
	Secondary school (*n* = 176) 396.45 ± 85.95			
Further trainings in other fields of medicine	Yes (*n* = 146) 422.92 ± 80.55	20.22 ± 9.44	−38.79 to 1.65	0.07
	No (*n* = 170) 402.69 ± 86.23			
Sex	Male (n = 17) 430.14 ± 82.56	18.23 ± 20.96	−23.00 to 59.46	0.39
	Female (*n* = 303) 411.91 ± 84.16			
Native language	German (n = 197) 417.71 ± 77.98	22.23 ± 19.94	−17.07 to 61.53	0.27
	Other (n = 17) 395.48 ± 89.00			

Table 3 presents correlations between the sociodemographic variables and levels of CHC.

**Table 3:** Multiple linear regression analysis adjusted for patient characteristics (dependent variable: person parameters)

	Regression coefficient	*P*-value
Training status	0.014	0.851
Country of birth	−0.160	0.060
Level of education	0.221	0.002
Basic profession	0.036	0.614
Further trainings in other fields of medicine	0.121	0.089
Sex	0.025	0.716
Native language	0.067	0.434
Age	−0.059	0.426
Professional experience	−0.162	0.136

## Discussion

This is the first study to survey the CHC of diabetes educators. The participants achieved a mean score of 409.84 (±88.10). Only the level of education seems to have had an influence on the level of CHC. Participants with a higher education degree had a slightly higher mean score of person parameters. However, the impact of slightly higher scores on the implementation of evidence-based practice principles in the participants’ working environment remains unclear. A total of 325 healthcare professionals completed the CHC test; 174 participants had already completed their training to become a certified diabetes educator and 151 were still in training. No differences were found between the two groups regarding the level of CHC. This is very likely, when reflecting the lack of implementation of evidence-based medicine methods within the 1800 h of training to become a certified diabetes educator, as skills in evidence-based medicine are needed to increase levels of CHC.

Some previous studies have used the CHC test to measure the CHC of healthcare professionals [19–22]. In all the trials, the feasibility of training programmes in evidence-based medicine was tested. The samples comprised secondary school students (grade 11), university students, patients, patient counselors, consumer representatives, healthcare professionals and physicians. The achieved mean person parameters in these trials ranged from 466 (±121) in untrained participants to 671.90 (± 51.38) in trained participants. Overall, diabetes educators in the present trial achieved competence scores slightly below those of untrained secondary school students or a self-selected group of patients and consumer representatives interested in evidence-based medicine [19–21]. These results underline the need for teaching strategies within the training curriculum for diabetes educators so that evidence-based medicine knowledge and skills can be increased.

Patients need unbiased information in order to make informed choices and to select the treatment that best fits individual patients’ needs, values and preferences. Diabetes educators are therefore requested to deliver evidence-based information about the benefits and harm of the treatment options. CHC are indispensable for conducting consultations based on principles of shared decision-making, and poor health competences have been identified as a major barrier to the implementation of shared decision-making in diabetes care [18, 23, 24, 25]. Training in methods of evidence-based medicine to increase levels of CHC has become an integral part of the education curricula for healthcare professionals [26, 27] and the results of systematic reviews on the effectiveness of training in evidence-based medicine show some positive impact on the CHC of healthcare professionals [28, 29, 30, 31].

The level of CHC achieved by diabetes educators in this trial is insufficient to provide evidence-based information for patients with diabetes mellitus. It is indispensable that essential core competences of evidence-based medicine are included in the training curriculum for diabetes educators. A minimum set of required competences that teaching and learning programs should cover has been developed and described [32]. By integrating these competences, diabetes educators should be able to recognize the rationale for evidence-based practice, ask the relevant clinical questions, acquire, appraise and interpret the evidence, involve patients in the decision-making process and evaluate the strategy used.

This study has several strengths. Approximately 200 diabetes educators complete their training in Germany every year. Our sample comprised 151 diabetes educators in training and so almost three-quarters of all trained diabetes educators in one year. Most studies which measured CHC did not apply validated instruments. Knowledge and skills in evidence-based medicine were often determined by self-assessment [29, 33]. In our study, we used the validated CHC test to measure levels of CHC.

The study also has limitations. The focus taken in our study on reading and understanding health information might have been too narrow to capture the wide range of cognitive and social skills that people need to make best use of health systems. The CHC test is strongly linked to the concept of evidence-based medicine. Therefore, other aspects of CHC, such as the ability to make the best use of the health system or communicative skills are barely covered by the CHC test. We did not collect data on how many diabetes educators were offered the test, because recruitment was also performed via announcement and email lists. Therefore, we don’t know if there are any differences in the characteristics between those who took the test and those who declined.

## Conclusion

The CHC of the healthcare professionals who participated in this trial are limited and the present training of diabetes educators is unlikely to have an impact on these competences. In order to integrate research evidence and patients’ values and preferences into the care of people with diabetes, core concepts of evidence-based medicine should be included into the curricula for diabetes educators.

## Data Availability

The datasets used and/or analyzed during the current study are available in an anonymised format from the corresponding author on reasonable request.

## Abbreviations

ADA: American Diabetes Association
ANOVA: Analysis of variance
CHC: Critical Health Competence
CHC test: Critical Health Competence test
EASD: European Association for the Study of Diabetes
SDM: Shared decision making
SPSS: Statistical Package for the Social Sciences
